# Carbohydrate limitation, but not exposure to the insecticide flupyradifurone, impairs wild bumblebee nest initiation

**DOI:** 10.1093/ee/nvag086

**Published:** 2026-08-20

**Authors:** Elena Couper Coombs, Christoph Grüter, Harry Siviter

**Affiliations:** School of Biological Sciences, University of Bristol, Bristol, UK; School of Biological Sciences, University of Bristol, Bristol, UK; School of Biological Sciences, University of Bristol, Bristol, UK

**Keywords:** flupyradifurone, bee decline, pollinator, sucrose concentration, bee nutrition, bee health

## Abstract

Loss of floral resources and pesticide exposure are key drivers of wild pollinator declines. Synergistic interactions between pesticides and poor nutrition may exacerbate individual effects, but how these anthropogenic stressors interact remains poorly understood, particularly across understudied life phases. Queen bumblebees in temperate regions emerge from hibernation in spring and attempt to initiate a colony. In agricultural environments, queens typically emerge during (i) a carbohydrate “hunger gap,” whereby nectar demand is higher than supply and (ii) during a time of year in which pesticide use is high. Here, we assessed the impact of carbohydrate limitation and exposure to the novel insecticide flupyradifurone on colony initiation of wild-caught bumblebee (*Bombus terrestris*) queens. Using a fully crossed experimental design, queens were exposed to a field-realistic concentration of flupyradifurone (1.6 ppm) at high (50% (w/w)) or low (15% (w/w)) sucrose concentrations. We found that bees provided with low-sucrose concentrations produced no eggs or larvae compared with 31.6% in the high concentration groups. Queens fed a lower concentration of sucrose also developed smaller ovaries and performed less nest initiation behaviors. We also found that exposure to flupyradifurone increased the activity of bumblebee queens, and that carbohydrate limitation and flupyradifurone synergistically reduced the likelihood of nest initiation behavior. These results demonstrate the importance of carbohydrate intake during the spring when queens are founding colonies, highlighting the need to consider not only floral diversity within environmental land management schemes but also floral quality.

## Introduction

Pollination services provided by bees are essential for the maintenance of natural ecosystems and sustainable agriculture, with an estimated 87.5% of flowering plants reliant on animal pollination (Klein et al. 2007, Ollerton 2017). Widely documented wild bee declines therefore threaten food security and wild ecosystems (Cameron and Sadd 2020, Zattara and Aizen 2021). Wild bee declines are being driven by a multitude of different anthropogenic stressors including loss of habitat (Potts et al. 2010, Goulson et al. 2015, Cameron and Sadd 2020). Intensive agricultural practices typically reduce the availability and quality of floral resources, limiting the nutritional landscape (Tscharntke et al. 2005, Pywell et al. 2011, Baude et al. 2016, Wright et al. 2024). Bees require nectar as a source of carbohydrates to fuel daily energy requirements, whereas pollen provides the protein for brood rearing (Carnell et al. 2020, Wright et al. 2024). The nutritional quality of nectar and pollen varies depending on plant species and environmental conditions (Vaudo et al. 2018, 2024, Filipiak et al. 2022). A foraging bee will try to maximize its efficiency by choosing flowers offering nectar (carbohydrate) with a higher nutritional value, but in landscapes with limited floral resources, bees may be carbohydrate restricted (Cnaani et al. 2006, Fowler et al. 2016, Brown and Brown 2020). Indeed, even in an environment with a high volume of low-quality nectar, carbohydrate intake can still be restricted, as bees are limited by their capacity to process water and so they cannot fully compensate by increasing consumption (Leach and Drummond 2018, Woodard et al. 2019, Gray et al. 2024). Consequently, even in an environment with a high number of nectar-poor floral resources (eg certain mass blooming crops), bees can be carbohydrate limited (Brown and Brown 2020, Linguadoca et al. 2021, Gray et al. 2024).

Intensive agriculture is also reliant on agrochemicals to control crop pests, and exposure to certain pesticides at field-realistic concentrations can have both lethal and sublethal impacts on bees (Goulson et al. 2015, Rundlöf et al. 2015, Siviter et al. 2018b, 2024, Tosi and Nieh 2019, Tosi et al. 2022, Fischer et al. 2023, Nicholson et al. 2024, Hicks et al. 2025). In the EU, the use of certain neonicotinoid insecticides (clothianidin, imidacloprid, thiacloprid, and thiamethoxam) is now restricted, which has increased the demand for replacement insecticides (Brown et al. 2016). Flupyradifurone (commercial formula Sivanto) is a butanolide insecticide that is chemically distinct from neonicotinoids, although with a similar mode of action, targeting insect nicotinic acetylcholine receptors (nAChRs) (Nauen et al. 2015). It is licensed for use on flowering and non-flowering crops (US EPA 2014, Nauen et al. 2015) and can have both lethal and sub-lethal consequences for bees at field-realistic levels. The pesticide can lower the reproductive output of both solitary (*Osmia bicornis* and *Xenoglossa pruinosa*) and social bees (*B. terrestris* and *B. impatiens*) and can also impair bee learning, memory, and foraging (Knauer et al. 2022, Siviter and Muth 2022, Fischer et al. 2023, Gray et al. 2024, Richardson et al. 2024, Rondeau and Raine 2024). Furthermore, exposure to Sivanto (chemical formulation containing flupyradifurone) can induce high levels of solitary bee mortality when used following label guidelines (Knauer et al. 2022, Siviter et al. 2024). While flupyradifurone exposure can detrimentally influence bees, our understanding of the non-target impacts of this pesticide is still limited, and we know little about how it interacts with other anthropogenic stressors.

Within agricultural environments, pollinators are typically exposed to multiple stressors simultaneously, and interactions between stressors may exacerbate the individual effects (Folt et al. 1999, Doublet et al. 2015, Siviter, et al. 2020a, 2021a). Interactions between stressors may be: (i) additive: the cumulative effect of multiple stressors is equal only to the sum of their individual effects, (ii) synergistic: the combined stressors have an effect larger than would be predicted for an additive effect, or (iii) antagonistic: the effect of combined stressors is smaller than predicted by the additive effect (Folt et al. 1999, Siviter et al. 2021a). Within a monoculture environment, the nectar of certain crops may contain a low concentration of sucrose (eg pear, oil seed rape), and so bees may increase the volume of nectar they consume, and, as a consequence, be exposed to higher doses of pesticides (Tong et al. 2019, Linguadoca et al. 2021, Gray et al. 2024). Likewise, sub-lethal pesticide exposure may have impacts on bee foraging, which leads to lower efficiency, and nutritional stress (Raine and Chittka 2008, Gill and Raine 2014, Siviter et al. 2018b, 2021b, Siviter and Muth 2022, Gray et al. 2024). While previous studies have reported additive and synergistic interactions between poor nutrition and pesticide exposure (Dance et al. 2017, Tosi et al. 2017, Leza et al. 2018, Tong et al. 2019, Stuligross and Williams 2020, 2021, Ingwell et al. 2021, Linguadoca et al. 2021, Knauer et al. 2022), there are major gaps in our understanding of how these key stressors influence pollinators at different stages of their lifecycle (Siviter and Muth 2020, Siviter et al. 2021a).

Bumblebees are used as a model system for assessing the impact of anthropogenic stressors on wild bees due to their importance as pollinators and their observed declines (Potts et al. 2010, Cameron et al. 2011, Goulson et al. 2015, Cameron and Sadd 2020, Ghisbain et al. 2024). In temperate regions, bumblebees have an annual life cycle including solitary and social phases. In the spring, solitary queens emerge from hibernation and attempt to find a nest site and initiate a colony. They are solitary until their first clutch of eggs develop into adults (Alford 1975, Goulson et al. 2005). This early life cycle stage is vitally important because death of the queen at this solitary stage results in the ceasing of all potential reproductive activity. Solitary queens also lack the social “buffer” of unexposed bees inside the nest, and are consequently vulnerable to anthropogenic stressors (Rundlöf et al. 2015, Sgolastra et al. 2019). Furthermore, the availability of nutritional resources can affect the nutritional status of the queen throughout her life (Arena and Sgolastra 2014, Woodard et al. 2019). However, our understanding of the impacts of anthropogenic stressors on bumblebees is largely based on evidence from worker bees taken from commercial colonies. Specifically, we know little about the impact of carbohydrate limitation and novel pesticide exposure on post-emergence bumblebee queens. This is of particular concern, given that queen emergence can coincide with a “hunger gap” period of low nectar availability (Timberlake et al. 2019, Jachuła et al. 2021) and high pesticide use (Ewald and Aebischer 2000). Similarly, while past research has assessed the impact of neonicotinoids on bumblebees queens (Baron et al. 2017a, 2017b, Leza et al. 2018, Sgolastra et al. 2018, Richardson et al. 2024) we know little about how alternative systemic insecticides influence the fecundity, behavior, and physiology of bumblebee queens.

Here, we assessed the individual and combined impact of carbohydrate limitation and the insecticide flupyradifurone on queen bumblebee (*Bombus terrestris audax*) colony initiation. In a fully crossed experimental design, we provided wild-caught queens with either low or high-quality sucrose solutions treated or untreated with flupyradifurone. We assessed the impact of these stressors on bee mortality, behavior, ovary development, and reproductive output. We hypothesized that a low-sucrose concentration diet would exacerbate the effects of insecticide exposure and result in a negative, synergistic impact on queen reproductive output.

## Methods

### Collection

Wild bumblebee queens (*Bombus terrestris*) were collected between March and April 2024 from a public garden managed by the University of Bristol (latitude: 51.457760, longitude: −2.602906). Individual queens visiting flowers were caught in nets and placed into individual collection vials in which they were transferred on icepacks to the laboratory. No insecticides are used in the public garden, but we cannot rule out the possibility that bees may have been exposed to pesticides in residential gardens surrounding the park prior to the experiment. However, any pesticide exposure will have been randomly distributed between experimental treatments (see below for details). We did not catch queens collecting pollen as this indicates an established nesting site (Tripodi and Strange 2019).

We collected faecal samples from queens in pots using 10-μl micropipettes and screened for parasites (*Vairimorpha* spp., *Crithidia bombi*, and *Apicystis bombi*) using an Olympus CH series microscope at 400× magnification (Baron et al. 2017b). Any infected queens were excluded from the experiment (Supplementary Table S1). All queens were re-examined for parasites post experiment, and parasitized bees were removed from the subsequent analysis (Supplementary Table S1, see below for details). Queens were housed in Perspex queen-rearing boxes (14 cm × 7.6 cm × 6.4 cm), in a climate-controlled walk-in chamber (26 °C, 60% relative humidity). The queens were provided with pollen pellets (2.5 g ± 0.5) and ad libitum sucrose treatments according to their relevant treatment group (see below, treatment administration). The pollen pellets were created by combining honeybee collected pollen (Koppert, Haverhill, United Kingdom), and 50% w/w sucrose.

### Treatment Administration

In a fully crossed experimental design, queens were randomly assigned to 1 of 4 treatment groups, consisting of: Control (50% w/w sucrose), Control (15% w/w sucrose), Pesticide (50% w/w sucrose), Pesticide (15% w/w sucrose). The experimenter (ECC) was blind to treatment throughout the entirety of the experiment.

#### Sugar Diet Treatments

Here, we defined a carbohydrate limited diet as access to a sucrose solution with a lower sugar concentration (15% w/w compared with 50% w/w). Optimal nectar concentrations for bumblebees range from 35% to 65% w/w (Harder 1986, Kim et al. 2011, Pamminger et al. 2019, Linguadoca et al. 2021) and vary across landscapes. Oilseed rape, a commonly grown crop exploited by bumblebees which flowers in the Spring, has a nectar concentration of approximately 30% at initial flowering, gradually reducing to 10% by the end of the flowering period (Cresswell and Osborne 2004, Pierre et al. 1999, Stanley et al. 2013).

Therefore, 15% w/w sucrose was considered a reasonable representation of low concentration diet; 50% w/w was chosen as the high concentration diet as this falls within the upper end of optimal concentrations and is standard for assessing pesticide toxicity (OECD 2017, Linguadoca et al. 2021). Although sucrose was provided ad libitum, carbohydrate consumption is still limited by the capacity of a bumblebee to process water (see above).

#### Pesticide Treatments

To make the pesticide solutions, flupyradifurone (PESTANAL, analytical standard 100 mg) was combined with water to create a stock solution. This was stored in a freezer at −20 °C to prevent degradation. Pesticide solutions were prepared by defrosting the stock solution and diluting it into the relative sucrose concentrations (15% w/w or 50% w/w) for a final pesticide concentration of 1.6 ppm (1.6 mg/kg) (Richardson et al. 2024). Although concentrations may reach as high as 4 ppm in oilseed rape nectar and up to 21 ppm in oilseed rape pollen, 1.6 ppm is considered a level of concern as a residue value for acute exposure by the US Environmental Protection Agency (European Food Safety Authority 2013, Glaberman and White 2014, Richardson et al. 2024). However, we acknowledge that our exposure regime is conservative, as bees may be exposed to higher concentrations of flupyradifurone depending on pesticide application (Glaberman and White 2014). Queens were provided with pesticide-treated syrup for 14 d, and untreated syrup for a further 14 d.

#### Queen Monitoring

We took daily recordings of queen mortality, egg presence, and nest behavior. Daily observations of each queen were made under red light, and we recorded a “behavioral snapshot” once per day. All behavioral recordings were taken at the same time (3 to 4 PM), to minimize the influence of circadian rhythm. Six behaviors were recorded: (i) actively moving, (ii) inactive, (iii) feeding, (iv) wax secretion, (v) incubation, (vi) brood antennation (see Supplementary Table S2 for detailed description). Wax secretion, incubation, and brood antennation were all considered to be behaviors directly associated with nesting (see below for analysis information).

Sucrose (15 or 50% w/w depending on treatment, see above) was weighed and replaced weekly for each nest. We also established 56 evaporative controls in total and used this to calculate average syrup loss for both of the concentrations (consumption–evaporation control). Pollen not containing eggs was replaced on days 3 and 7 of each week (Siviter et al. 2018a, 2020b). Queens that died during the experiment were frozen. At the end of the 4 wk, all nests were frozen.

### Dissection

Post experiment, all nests were dissected. We recorded (i) the presence or absence of brood, (ii) the number of eggs, and (iii) the number of larvae. The thorax width of all queens was measured using digital calipers. We dissected the queens in phosphate-buffered saline using a GXOptical dissection microscope at 10× to 30× magnification. The abdomen was checked for *Sphaerularia bombi* larvae and internal mites (*Locustacaris buchneri*), and samples from the malpighian tubules, hindgut, and fat body were screened for *Crithidia bombi*, *Apicystis bombi*, and *Vairimorpha* spp. using an Olympus CH series microscope at 400× magnification (see above). Infected queens were excluded from subsequent analysis (Supplementary Table S1). Ovaries were dissected out and checked for the presence of terminal oocytes. In cases when ovaries had developed, we measured the length of each oocyte at 20× magnification with the use of a Leica M205C stereo dissection scope and GXCapture software.

### Statistical Analysis

We applied an information-theoretic model selection approach using Akaike Information Criterion (AICc) values to evaluate model fit. For each analysis, we constructed a full model including all relevant factors and any random effects (see below subsections for individual factors and random factors where relevant and see Supplementary Table S3 for model outputs). This model was compared to every ecologically relevant subset of the model and a null model containing only the intercept. Model selection was conducted by choosing the models with the lowest AICc values. In cases when multiple models have ΔAIC < 2 we used model averaging (Supplementary Table S3).

Depending on the dependent variable (see below, Supplementary Tables S3 and S4 for full list), we used Cox proportional hazards models, linear mixed-effects models, generalized linear models, and generalized linear mixed-effects models. In all relevant models, we assessed whether synergistic interactions occurred between pesticide exposure and sucrose concentration. To do this, pesticide exposure, sucrose concentration, and their interaction were all included in all full models, and the output of the interaction was used to determine whether the interaction was additive, synergistic or antagonistic (see above for definitions). Bee was included as a random effect in cases where repeated measures were taken. All models are reported in full in Supplementary Table S3. We used R (version 4.3.3) and the *MuMIn*, *lme4*, and *survival* packages to conduct our analysis (Bates et al. 2015, Therneau 2018, Bartoń 2024, R Core Team 2024).

#### Survival

We analyzed survival data using a mixed-effects Cox proportional hazards model. Pesticide exposure, sucrose concentration, their interaction, bee size, and date of capture were all included in the full model. Bees that died during the experiment were removed from all subsequent analyses.

#### Brood

We assessed the impact of pesticide exposure and sucrose concentration on the timing of brood production and the total brood number (combined number of eggs and larvae). Since no brood was produced in the low-sucrose concentration groups, these were excluded from all models. To analyze the time it took for brood production to occur, we used a mixed-effects Cox proportional hazards model including bee size, date caught, sucrose concentration, pesticide exposure, and the interaction between pesticide exposure and sucrose concentration. Brood number was analyzed using a Poisson distributed generalized linear mixed-effects model.

#### Ovaries

We analyzed the effect of treatment on the presence and size of terminal oocytes. In the full model, we included date of capture, bee size, pesticide exposure, sucrose concentration, and the interaction between pesticide exposure and sucrose concentration. We analyzed the presence of terminal oocytes using a generalized linear mixed-effects model with a binomial distribution. Both the maximum terminal oocyte length and the size of individual terminal oocytes were analyzed using a linear mixed-effects model, with bee included as a random factor in the analysis of individual oocytes.

#### Behavior

Both nesting behaviors (wax secretion, incubating, brood antennation, see Supplementary Table S2) and activity levels (bees performing any behavior other than inactivity were deemed active, see Supplementary Table S2) were analyzed using a generalized linear mixed-effects model with a bino­mial distribution. In the full model, we included bee size, date of capture, day, pesticide exposure, sucrose concentration, and the 3-way interaction between pesticide, sucrose concentration, and day. Bee was included as a random factor.

#### Sucrose

Both weekly syrup and sucrose consumption data were adjusted for evaporation and analyzed using a linear mixed-effects model with bee included as a random factor. All data points with recorded spillages were removed (*n* = 2). In the full model, we included pesticide, sucrose concentration, week, bee size, and date as well as all levels of the interaction between pesticide, week, and sucrose concentration. Bee was included as a random factor.

The model outputs for all dependent variables are given in full in Supplementary Table S4.

## Results

### Mortality

We found no significant effect of carbohydrate limitation or pesticide exposure on queen mortality (Supplementary Fig. S1; pesticide: parameter estimate PE = −0.23, 95% confidence interval [CI] = −0.91 to 0.46; sucrose concentration: PE = 0.48, CI = −0.09 to 1.05) and no interaction effect (pesticide * sucrose concentration: PE = 0.28, CI = −0.60 to 1.15). Queens caught at a later date were significantly more likely to survive (Supplementary Fig. S2; capture date: PE = 0.03, CI = 0.01 to 0.05).

### Brood

**Fig. 1.**
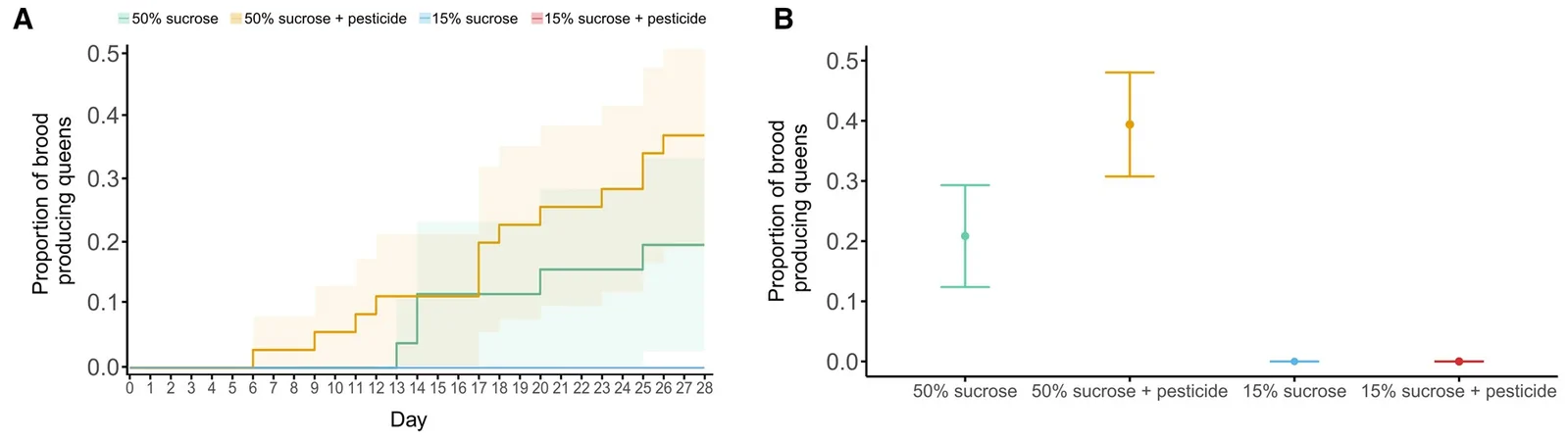
Brood production: A) Kaplan–Meier plot illustrating the proportion of queens (±SE) with brood by each day of the experiment (50% sucrose, *n* = 24; 15% sucrose, *n* = 33; 50% sucrose + pesticide, *n* = 20; 15% sucrose + pesticide, *n* = 20). B) Proportion of queens (±SE) that produced brood during the experiment across 4 treatment groups: 50% sucrose (*n* = 24), 15% sucrose (*n* = 19), 50% sucrose + pesticide (*n* = 33), 15% sucrose + pesticide (*n* = 20). None of the low-sucrose groups produced brood.

None of the queens in the low-sucrose concentration groups produced brood, compared to 31.6% from the high concentration groups (Fig. 1), indicating that carbohydrate limitation can strongly reduce nest initiation. Of the bees provided with 50% sucrose, pesticide exposure had no significant effect on brood number, but bee size was a significant predictor, with smaller bees producing slightly less brood (pesticide: ΔAICc >2 [meaning that pesticide was not selected in the top models, see Supplementary Table S3]; thorax width: Supplementary Fig. S3; PE = 0.98, CI = 0.25 to 1.71).

### Ovaries

**Fig. 2.**
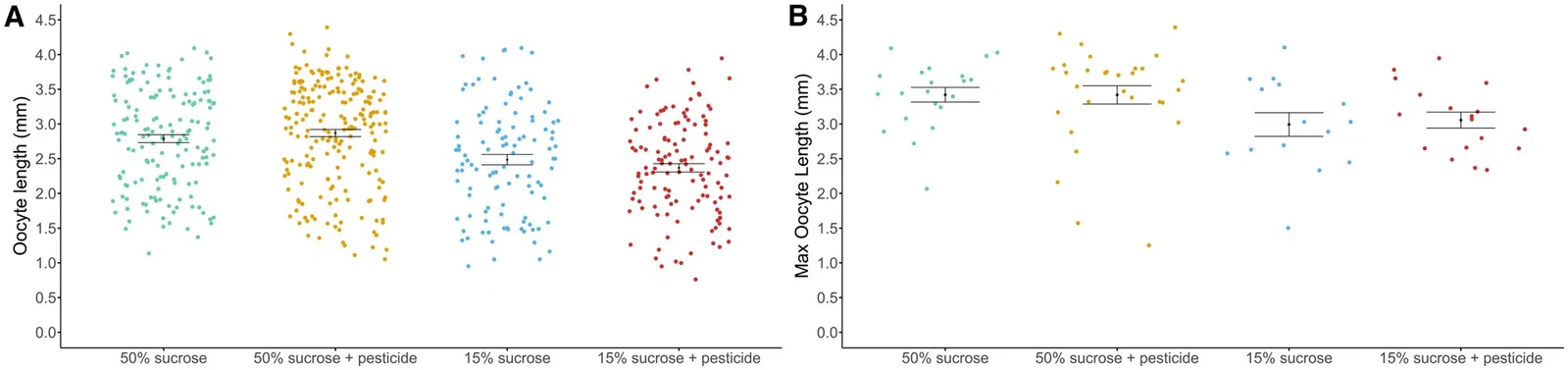
Oocyte size: A) Length of terminal oocytes (mm ± SE), with each data point representing a single oocyte. Queens are from 4 treatment groups: 50% sucrose (*n* = 21 bees, *n* = 158 oocytes), 15% sucrose (*n* = 15 bees, *n* = 111 oocytes), 50% sucrose + pesticide (*n* = 30 bees, *n* = 213 oocytes), 15% sucrose + pesticide (*n* = 18 bees, *n* = 127 oocytes). B) Length of the largest terminal oocyte (mm ± SE) of queens from 4 treatment groups: 50% sucrose (*n* = 24, *n* = 21 ovaries), 15% sucrose (*n* = 19, *n* = 15 ovaries), 50% sucrose + pesticide (*n* = 33, *n* = 30 ovaries), 15% sucrose + pesticide (*n* = 20, *n* = 18 ovaries).

Neither pesticide exposure nor sucrose concentration influenced the presence or absence of developed ovaries (pesticide: PE = 0.16, CI = −0.67 to 0.98; sucrose concentration: PE = −0.10, CI = −0.76 to 0.57; pesticide*sucrose concentration: ΔAICc >2), but queens in the low-sucrose concentration groups produced oocytes 17% smaller than those in high-sucrose concentration groups (Fig. 2A; sucrose concentration: PE = −0.41, CI = −0.66 to −0.16). The maximum length of terminal oocytes was also 12.98% smaller in low-sucrose concentration groups (Fig. 2B; sucrose concentration: PE = −0.40, CI = −0.67 to −0.13). These data confirm that sucrose limitation can limit queen ovary development. There was no effect of pesticide on oocyte length and no significant interaction between pesticide and sucrose concentration (max oocyte length: pesticide: ΔAICc >2; pesticide*sucrose concentration: ΔAICc >2; individual oocyte length: pesticide: ΔAICc >2; pesticide*sucrose concentration: ΔAICc >2).

### Behavior

**Fig. 3.**
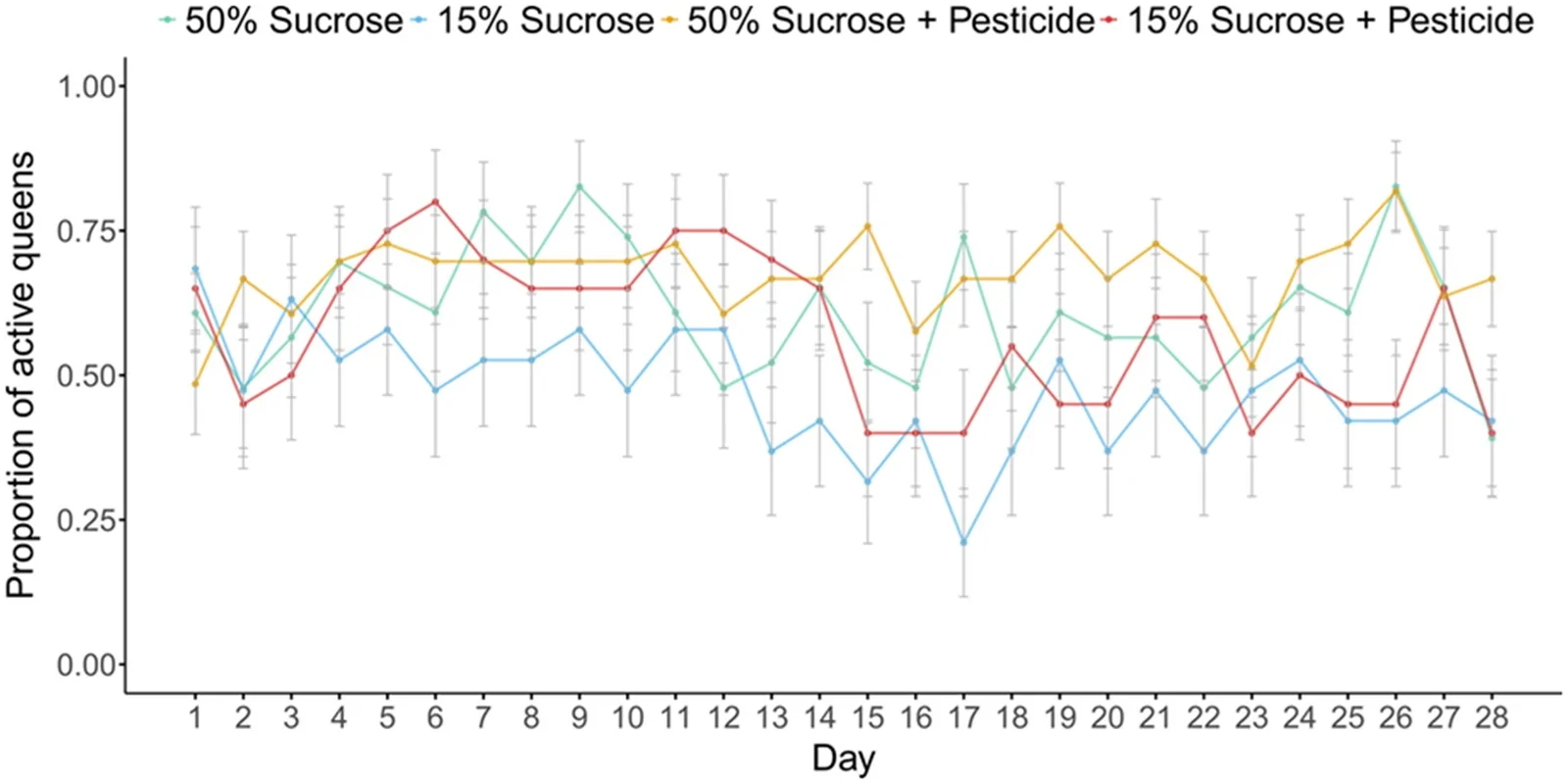
Queen activity: proportion (±SE) of bees active over the course of the experiment from 4 treatment groups: 50% sucrose (*n* = 24), 15% sucrose (*n* = 19), 50% sucrose + pesticide (*n* = 33), 15% sucrose + pesticide (*n* = 20).

Sucrose concentration influenced the proportion of queens that were active during the behavioral observation. We observed 23.7% less activity in low-sucrose concentration groups (Fig. 3), although carbohydrate limitation in isolation was not a significant predictor of activity (sucrose concentration: PE = −0.04, CI = −0.48 to 0.40). However, the interaction between day and sucrose concentration was significant, indicating that queens in the 15% (w/w) groups became less active as the experiment progressed (Fig. 3; day * sucrose concentration: PE = −0.03, CI = −0.05 to −0.01). In contrast, exposure to flupyradifurone significantly increased the proportion of active queens by 30.2% (Fig. 3; pesticide: PE = 0.39, CI = 0.07 to 0.71), although we found no evidence of an interaction between sucrose concentration and pesticide or any temporal changes with day (pesticide * sucrose concentration; pesticide * day; pesticide * sucrose concentration * day, ΔAICc >2).

**Fig. 4.**
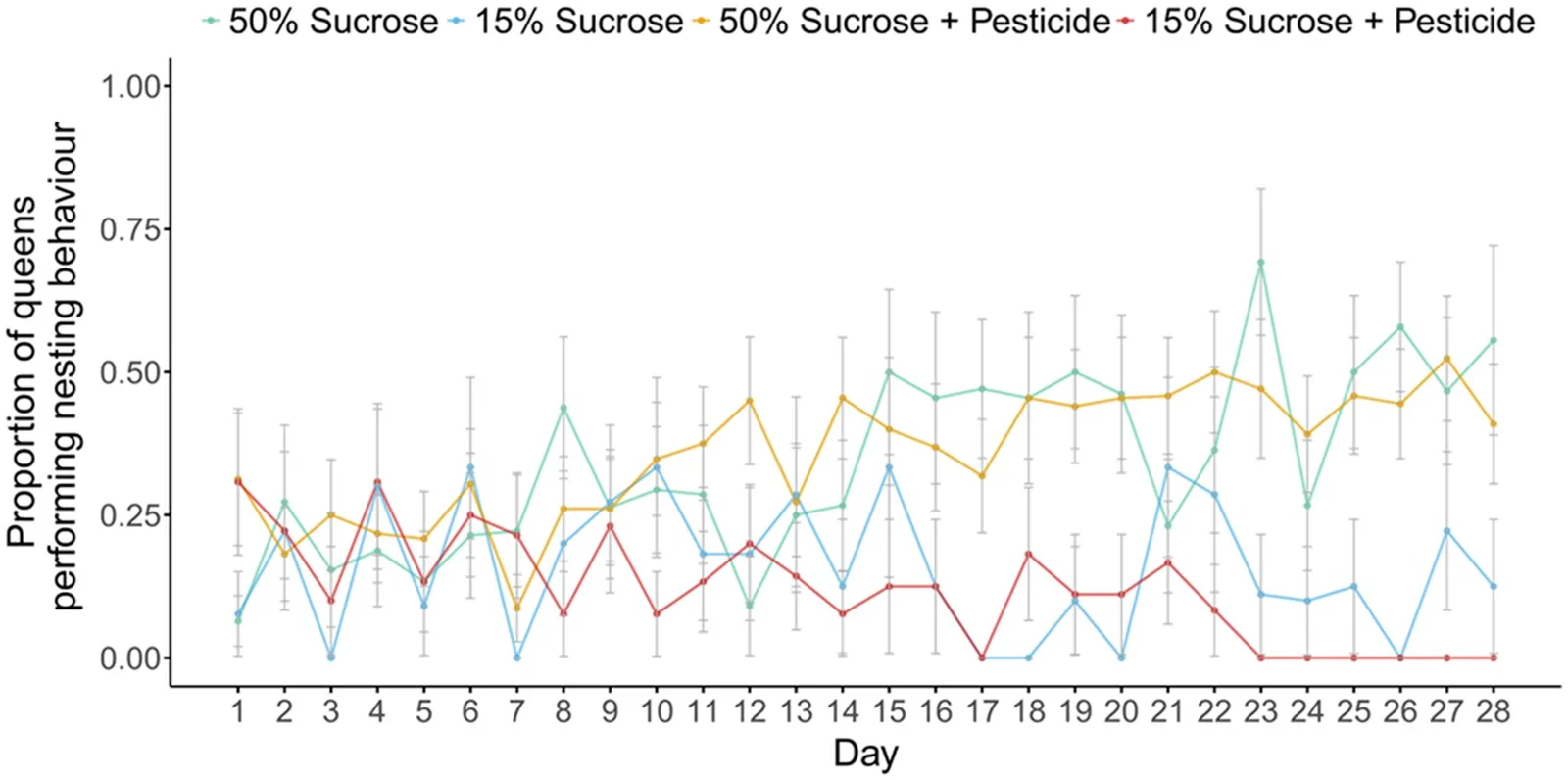
Nesting behavior: proportion (±SE) of bees exhibiting nest initiation behaviors (wax secretion, incubating, brood antennation) on each day of the experiment from 4 treatment groups: 50% sucrose (*n* = 24), 15% sucrose (*n* = 19), 50% sucrose + pesticide (*n* = 33), 15% sucrose + pesticide (*n* = 20).

In relation to nesting behavior (including wax secretion and incubation behavior, see Supplementary Table S2), we found a significant 3-way interaction between day, sucrose concentration and pesticide presence, indicating that low-sucrose concentrations and pesticide exposure synergistically reduced the likelihood of nesting behavior with time (Fig. 4; day*sucrose concentration*pesticide: PE = −0.09, CI = −0.18 to −0.01). We also found a significant interaction between sucrose concentration, and day, with bees in the 15% (w/w) sucrose groups being 17.9% less likely to perform behaviors associated with nest initiation, a trend that became stronger as the experiment progressed (Fig. 4; sucrose concentration: PE = 0.24, CI = −0.97 to 1.44; sucrose concentration and day interaction: PE = −0.08, CI = −0.13 to −0.02). Pesticide alone was not a significant predictor of nesting behavior, nor was the 2-way interaction between low-sucrose and pesticide without the day factor (pesticide: PE = 0.17, CI = −0.81 to 1.16; pesticide*sucrose concentration: PE = 0.41, CI = −1.21 to 2.04). Bees caught at a later date were also significantly less likely to perform nesting behaviors (Supplementary Fig. S4; PE = −0.07, CI = −0.11 to −0.04), and as predicted, we found an overall increase in nesting behavior with time from the start of the experiment (Fig. 4; day: PE = 0.07, CI = 0.04 to 0.10).

### Sucrose

**Fig. 5.**
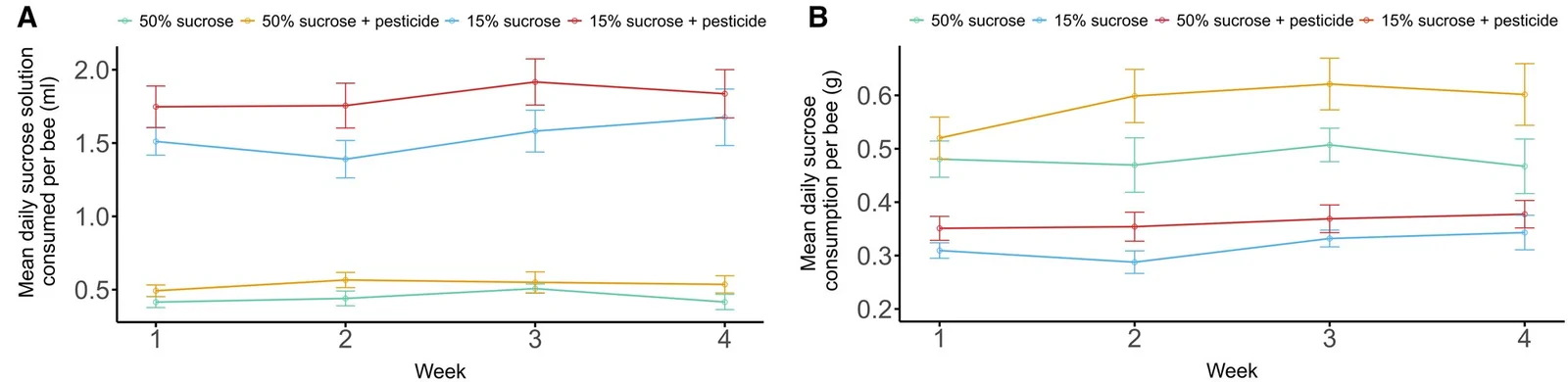
Sucrose consumption: A) Mean (±SE) sucrose solution consumed daily (g) by queens over 4 wk. Queens were assigned to 4 treatment groups: 50% sucrose (*n* = 24), 15% sucrose (*n* = 19), 50% sucrose + pesticide (*n* = 33), 15% sucrose + pesticide (*n* = 20). B) Mean (±SE) grams of sucrose consumed daily over 4 wk by queens in 4 treatment groups: 50% sucrose (*n* = 24), 15% sucrose (*n* = 19), 50% sucrose + pesticide (*n* = 33), 15% sucrose + pesticide (*n* = 20).

Low-sucrose concentration significantly increased daily consumption of sucrose solution (Fig. 5A; sucrose concentration: PE = 1.19, CI = 1.05 to 1.33), which will have in turn increased pesticide exposure. But, despite consuming a great volume of sucrose solution, we found that bees in the low-sucrose concentration groups consumed significantly less sucrose (g) than those in the high concentration groups (Fig. 5B; sucrose concentration: PE = −0.19, CI = −0.25 to −0.13). This confirms that queens in the low-sucrose concentration group were carbohydrate limited. We did not find an effect of pesticide or any interaction between pesticide exposure and sucrose concentration on the amount of sucrose consumed in grams or volume (Supplementary Table S4).

## Discussion

In the Anthropocene, wild bees are often simultaneously exposed to pesticides and poor-quality foraging resources (Goulson et al. 2015, Cameron and Sadd 2020, Siviter and Muth 2020). We found that none of the queens provided with a low-quality sucrose concentration initiated a nest. The queens also demonstrated less nesting behavior and had smaller terminal oocytes compared to queens fed on a higher sucrose concentration. Exposure to flupyradifurone did not influence nest initiation, although pesticide exposure did increase queen activity. We also observed a synergistic interaction between pesticide exposure and sucrose concentration on nesting behavior over time. These results indicate that carbohydrate limitation in the spring, as a consequence of poor floral resources, will likely have negative impacts on bumblebee nest initiation, which could have population level consequences for this important pollinator.

We found that queens in the low-sucrose concentration groups produced no brood, compared to 31.6% in high-sucrose concentration groups. Previous studies have linked limited floral diversity with poor colony health and survival (Goulson et al. 2002, Jha and Kremen 2013, Pasquale et al. 2013, Leza et al. 2018, Klaus et al. 2021). The nutritional qualities of plants can vary between species and environmental conditions (Vaudo et al. 2018, 2024, Filipiak et al. 2022). The quality of certain nutrients in pollen, such as the sterol concentration, can impact colony fitness (Vanderplanck et al. 2014, Moerman et al. 2016, 2017, Vaudo et al. 2018, 2024, p. 202, Castle et al. 2022, Filipiak et al. 2022), and poor carbohydrate nutrition can limit the health and survival of honeybees and bumblebees (Tosi et al. 2017, Tong et al. 2019, Woodard et al. 2019, Linguadoca et al. 2021, Quinlan et al. 2023, Watrobska et al. 2024). Our results demonstrate that sucrose concentration is a key determinant of queen colony initiation in *B. terrestris*. Queens in the low-sucrose concentration groups consumed a smaller amount of sucrose than those in the high-sucrose concentrations groups, despite consuming 240.1% more solution. This demonstrates that they were not able to fully compensate for the low-quality nectar (Brown and Brown 2020). In addition, queens in the low-sucrose concentration groups had terminal oocytes 17% smaller than those in the high nutrition groups. They were also 17.9% less likely to perform behaviors associated with nesting, providing a functional mechanism behind the observed reduction in brood production. These results are concerning as in the United Kingdom, early spring (March–April) is when *B. terrestris* emerge from hibernation and start to initiate colonies. This time of year aligns with a “hunger gap” period in UK agricultural sites, whereby the demand for floral nectar, is higher than the available supply (Timberlake et al. 2019). One of the limitations of our study is that the nutritional manipulation is limited to sucrose concentration. The UK “hunger gap” is not driven by lower sucrose concentration per floral unit, but rather total sugar production across the environment (Timberlake et al. 2019). Nevertheless, our results indicate that colony initiation during this key life stage may be significantly impaired by carbohydrate limitation. Furthermore, under field conditions, bees will have to fly further in a low-nectar environment and so our results here may be considered conservative. Assessing the landscape impact of carbohydrate limitation on other wild bee species, including solitary bees, is an important area of future research (Burkle and Irwin 2009, Vaudo et al. 2015, Woodard and Jha 2017).

We found no evidence to indicate that flupyradifurone exposure impaired bumblebee nest initiation at the tested concentration, although flupyradifurone did significantly increase queen activity levels. Previous studies have observed hyperactivity in bees exposed to flupyradifurone, alone and in combination with another insecticide (Tosi and Nieh 2019, Rondeau and Raine 2024). Hyperactivity is a behavioral response characterized by increased activity and erraticism which depletes energy reserves and can impair bee foraging, flight, and nest-building abilities (Tong et al. 2019, Tosi and Nieh 2019, Anderson and Harmon-Threatt 2021, Rondeau and Raine 2024). However, contrary to our expectations, we found no evidence that flupyradifurone in isolation impaired queen nest initiation. This is likely due to the pesticide concentration tested (1.6 ppm) (US EPA 2014). The concentrations of flupyradifurone can reach up to 4.3 ppm in the nectar and honey stomachs of bees foraging on oilseed rape, and concentrations as high as 21 ppm have been found in oilseed rape pollen (US EPA 2014). A recent study found that flupyradifurone exposure at 1.6 ppm resulted in a lower total larval mass produced by *B. impatiens* microcolonies, but exposure to flupyradifurone at a 4 ppm dose resulted in a much more severe reduction in larval mass (97.9%) (Richardson et al. 2024). Other studies exposing bees to flupyradifurone concentrations of 4 ppm or higher have observed sub-lethal impacts on bee learning performance, flight ability and thermoregulation (Hesselbach and Scheiner 2019, Tong et al. 2019, Siviter and Muth 2022, Fischer et al. 2023, Gray et al. 2024). Currently, flupyradifurone is licensed for use on bee-visited crops during flowering, resulting in bees being exposed to high concentrations of the insecticide (US EPA 2014). These data and those from previous research (Hesselbach and Scheiner 2019, Tong et al. 2019, Siviter and Muth 2020, Knauer et al. 2022, Fischer et al. 2023, Gray et al. 2024, Siviter et al. 2024) provide support for limiting the use of flupyradifurone to non-flowering crops as a precautionary measure to reduce the risk of pollinator exposure (Siviter et al. 2024).

Synergistic interactions occur when exposure to multiple stressors exacerbates individual effects (Folt et al. 1999, Siviter et al. 2021a). Nutritional stress can exacerbate the negative effects of pesticide exposure (Tosi et al. 2017, Tong et al. 2019, Linguadoca et al. 2021, Knauer et al. 2022). Floral resources that are low in quality (eg low-sucrose concentration) can increase pesticide exposure as bees have to increase their consumption in order to attempt to meet their nutritional requirements (Tosi et al. 2017, Tong et al. 2019, Linguadoca et al. 2021, Knauer et al. 2022). Equally, a high-quality diet has been shown to limit the impact of pesticide exposure across multiple species by upregulating detoxification genes (Barascou et al. 2021, Castle et al. 2022, 2023). We observed a synergistic reduction in nesting behaviors, such as wax secretion and incubation (Supplementary Table S2), when queens were carbohydrate limited and exposed to the insecticide flupyradifurone. This interaction was mainly driven by wax secretory behaviors, due to the lack of brood in the low-sucrose concentration groups. In the low-sucrose concentration groups, queens consumed a higher amount of sucrose solution, consequently ingesting 107% more pesticide than queens in the high-sucrose concentration group (Tong et al. 2019, Linguadoca et al. 2021, Gray et al. 2024). Carbohydrate limitation may have also limited the ability of the bees to metabolize the pesticide, resulting in a lower tolerance to exposure (Tosi et al. 2017, Castle et al. 2023). However, we found no interaction between pesticide and sucrose concentration on any other measured variable, likely due to the conservative pesticide concentration tested (see above) (Richardson et al. 2024). Other studies have found both additive (Dance et al. 2017, Stuligross and Williams 2020, Gray et al. 2024) and synergistic (Tong et al. 2019, Ingwell et al. 2021, Linguadoca et al. 2021, Knauer et al. 2022) effects of nutritional stress and pesticide exposure on bees, highlighting a need for interactive effects between nutrition and pesticides to be considered within pesticide risk assessment (Siviter and Muth 2020, Linguadoca et al. 2021, Siviter et al. 2023).

In the United Kingdom, an estimated 97% of flower rich grasslands have been lost since the 1930s (Fuller 1987), and nectar production fell by 32% between 1930 and 1978 (Baude et al. 2016). Despite a 25% increase in nectar provision in Great Britain from 1998 to 2007, nectar production and diversity in the United Kingdom remains low relative to pre-1930 levels (Baude et al. 2016). Provisioning diverse floral resources has been shown to support wild bee populations, but our results add to growing evidence that plant quality and phenology should also be taken into account, particularly in sparse arable landscapes (Pierre et al. 1996, Redpath et al. 2010, Pywell et al. 2011, Wood et al. 2015, Carvell et al. 2017, Vaudo et al. 2018, 2024, Timberlake et al. 2019, Filipiak et al. 2022). The United Kingdom has transitioned away from the EU’s Common Agricultural Policy framework post-Brexit, adapting and expanding some of these policies to form part of the planned Environmental Land Management (ELM) schemes, including a modified Countryside Stewardship scheme and new Sustainable Farming Incentives which pay farmers for environmental goods and services, such as wildflower plantings and hedgerow maintenance (DEFRA 2023). Some ELM schemes pay farmers to establish blocks or strips of flowering plants, including species such as oxeye daisy (*Leucanthemum vulgare*), musk mallow (*Malva moschata*), or yarrow (*Achillea millefolium*) (DEFRA 2023). There are limited data available on the nectar concentrations of many of these plant species, but concentrations can reach as low as 13.1% in birds-foot trefoil (*Lotus Corniculatus*) and as high as 45% in sainfoin (*Onobrychis viciifolia*) (DeGrandi-Hoffman and Collison 1982, Farkas et al. 2007, DEFRA 2023). Bumblebees prefer floral resources with nectar concentrations of >50% sucrose (Bailes et al. 2018, Brown and Brown 2020), and while acknowledging that nectar concentration is highly variable across different environmental variables, including plants with both high nectar concentrations and early flowering time within environmental stewardship schemes would support solitary queens attempting to initiate colonies (Timberlake et al. 2019). Future research should focus on quantifying the nectar concentrations of flowers in agricultural environments so that agricultural schemes can tailored to support bumblebee queens at this vulnerable phase in their life cycle.

Habitat loss is one of the main drivers of bumblebee declines globally (Baude et al. 2016, Carvell et al. 2017). Despite initiatives by policy makers to mitigate pollinator declines, several species are range retracting and increasingly vulnerable to extirpation or extinction (IUCN 2024). Our results demonstrate that carbohydrate limitation can have a severe impact on colony initiation, a lifecycle stage which acts as a bottleneck for colony success (Arena and Sgolastra 2014, Rundlöf et al. 2015, Carvell et al. 2017, Woodard et al. 2019). These results show that conservation initiatives that are designed to promote pollinator health should incorporate not only floral diversity but also floral resource quality in terms of nectar production. Land management schemes that promote high-quality nectar resources during spring “hunger gap” periods will likely have a disproportionately important role in reversing bumblebees declines and safeguarding pollination services (Timberlake et al. 2019).

## Supplementary Material

nvag086_Supplementary_Data

## Data Availability

All data can be found on Open Science Framework (https://osf.io/zkp7f/overview).
