# Supplementary material for: Carbohydrate limitation, but not exposure to the insecticide flupyradifurone, impairs wild bumblebee nest initiation
Source: Environ Entomol. 2026 Aug 20;55(4):nvag086. doi: 10.1093/ee/nvag086 (PMC13493054; doi:10.1093/ee/nvag086)
Supplement: Supplementary file 1 [file nvag086_supplementary_data.docx]

**Carbohydrate limitation, but not exposure to the insecticide flupyradifurone, impairs wild bumblebee nest initiation.**

*Elena Couper Coombs, Christoph Grüter, Harry Siviter**

School of Biological Sciences, University of Bristol, 24, Tyndall Avenue, Bristol, BS8 1TQ, UK

*Corresponding author:

Harry Siviter

School of Biological Sciences,

University of Bristol,

24 Tyndall Avenue,

Bristol, BS8 1TQ, UK

[harry.siviter@bristol.ac.uk](mailto:harry.siviter@bristol.ac.uk)

Supplementary Materials

**Table S1:** Summary of collection and infection data for *Bombus terrestris* queens. Faecal samples were screened for parasites under a microscope prior to the experiment, any infected queens were excluded from the experiment. Post-experiment, samples from the hindgut, malpighian tubules and fat bodies were also screened for infection (see methods for details), and again infected queens were excluded from subsequent analyses.

**Table S2**: Ethogram of behaviours exhibited by *Bombus terrestris* queens during observation.

| **Behaviour** | **Description** |
| --- | --- |
| Actively moving | Queen is moving. |
| Inactive | Queen is still. |
| Sucrose feeding | Queen is feeding on sucrose solution. |
| Waxing | Queen is holding her abdomen flattened against the ground and is actively pulsating (Heinrich, 1974). |
| Incubation | Queen is perched on top of her brood and has flattened her abdomen. Abdomen is often pulsating (Heinrich, 1974; Sarro Gustilo et al., 2023). |
| Brood antennation | Queen is perched on top of her brood and is moving her antenna over the wax (Sarro Gustilo et al., 2023). |

**Table S3:** Output of candidate models used in each analysis. Only models with ΔAICc <2 were included in model averaging, top 8 presented here (for full models see statistical analysis). Sucrose concentration (50% or 15% sucrose), pesticide (presence or absence), date of queen capture, day (days into the experiment) and bee size (thorax width) were included. Models in bold were included in model averaging.

**Table S4:** Parameter estimates, 95% confidence intervals and standard error derived from averaging the top models. Those with 95% confidence intervals that did not include zero are highlighted in bold.

**Figure S1 Queen survival:** Kaplan-Meier plot demonstrating the proportion of queens surviving (±SE) over the course of the experiment from four treatment groups: high nutrition (n = 46), low nutrition (n= 49), high nutrition + pesticide (n = 47), low nutrition + pesticide (n = 51).

**Figure S2 Queen survival in relation to capture date:** Proportion (± SE) of surviving queens (n = 96) plotted against the date on which they were caught.

**Figure S3 Bee size against brood number:** number of brood (eggs and larvae) produced by individual queens plotted against queen thorax width (mm) (n = 96).

**Figure S4 Brood care against date caught:** proportion (± SE) of queens from each date of capture which exhibited brood care behaviour at least once over the course of the whole experiment (n=96).

References

Heinrich, B. (1974). Pheromone Induced Brooding Behavior in *Bombus vosnesenskii* and *B. edwardsii* (Hymenoptera: *Bombidae*). *Journal of the Kansas Entomological Society*, *47*(3), 396–404.

Sarro Gustilo, E., Fisher, K., & Woodard, S. H. (2023). Parental care behaviour in bumble bee queens is tightly regulated by the number of helpers in incipient nests. *Animal Behaviour*, *203*, 241–250. https://doi.org/10.1016/j.anbehav.2023.07.009
